# Nerve Injury Following Birth, the Role of Epidural Analgesia—A Protocol for a Scoping Review

**DOI:** 10.1111/aas.70313

**Published:** 2026-08-04

**Authors:** Louise Mathilde Loft Sakhat, Amanda Prip Dohn, Bo Biering‐Sørensen, Kenneth Geving Andersen, Line Thellesen, Kim Wildgaard

**Affiliations:** ^1^ Anaesthesia Critical and Emergency Care Science Unit and Department of Anaesthesiology Herlev Hospital Herlev Denmark; ^2^ Department of Clinical Medicine, Faculty of Health and Medical Science University of Copenhagen Copenhagen Denmark; ^3^ Movement Disorder Clinic, Neuropathic Pain and CRPS Clinic Spasticity Clinic, Movement Disorder and Pain Research Center Spasticity Clinic, Movement Disorder and Pain Research Center Rigshospitalet Glostrup Denmark; ^4^ Neurological Department Copenhagen University Hospital, Rigshospitalet Glostrup Copenhagen Denmark; ^5^ Department of Anesthesia and Intensive Care Hvidovre Hospital, University of Copenhagen Copenhagen Denmark; ^6^ Department of Pregnancy and Childbirth Herlev University Hospital Herlev Denmark

## Abstract

**Background:**

Epidural analgesia is widely used during labor, and postpartum nerve injury is an uncommon but clinically relevant outcome. Neurological symptoms after childbirth may be related to obstetric or anesthetic factors. Studies vary considerably in how postpartum nerve injury is identified, defined, assessed, and followed, limiting comparability and interpretation of findings.

**Objective:**

To map the methodological approaches used to study postpartum nerve injury following epidural analgesia, including case identification strategies, diagnostic assessment methods, follow‐up procedures, and reporting of obstetric and anesthetic attribution.

**Methods:**

A scoping review will be conducted in accordance with the PRISMA‐ScR framework. Studies reporting postpartum nerve injury in relation to epidural procedures will be identified through systematic database searching. Data will be charted on study design, exposure definitions, strategies for etiological attribution, outcome ascertainment, and follow‐up methods.

**Discussion:**

The scoping review aims to map how postpartum nerve injury has been defined, identified, assessed, and followed in the literature, as well as clarify methodological patterns and inconsistencies. The findings may inform future research by highlighting variation in methodology, reporting practices and terminology. Thereby indicating areas where more consistent approaches to neurological assessment, exposure reporting, and attribution of obstetric and anesthetic factors are needed.

## 1 Introduction

### 1.1 Background

Epidural analgesia is a central component of pain management during labor and is widely used in modern obstetric practice [1, 2]. Increased use of epidural analgesia has led to growing interest in maternal outcomes beyond analgesia effect, including postpartum nerve injury [3]. Nerve injury refers to structural or functional impairment of a peripheral nerve, nerve root, or plexus. Symptoms can present with weakness, sensory loss, altered reflexes and difficulty completing physical tasks [4].

Neurological symptoms after childbirth may occur even in uncomplicated vaginal deliveries and can be related to obstetric factors such as prolonged labor, fetal head compression, or maternal positioning during delivery [2, 5]. Affected nerves often involve peripheral nerves of the lumbosacral plexus, commonly the femoral, lateral femoral cutaneous or peroneal nerves [5, 6]. Neuraxial anesthesia may represent an additional contributing factor to the development of nerve injury.

Serious neuraxial complications related to anesthesia are uncommon, and most postpartum nerve injuries are transient [7, 8]. In the literature, the terms postpartum nerve injury, and neurological symptoms are used with varying definitions, ranging from patient‐reported sensory disturbances to clinically verified peripheral nerve deficits.

Several studies have examined the frequency, severity, and recovery patterns of postpartum nerve injury [3, 9]. Limited clarity remains regarding how postpartum nerve injuries are identified, assessed, and monitored across studies. Considerable variations are seen in detection methods, timing of assessment, definitions of nerve injury, and follow‐up procedures. Reporting of inclusion pathways and recruitment strategies is often incomplete [3, 8]. Methodological differences across studies make it difficult to determine whether reported variation in incidence reflects actual differences in risk or differences in study design.

An overview of methodological approaches used to study postpartum nerve injury following epidural analgesia is therefore needed.

### 1.2 Rationale and Objectives

Differences in study design, definitions, and assessment strategies are reported across studies. Variation in methodology limits comparability and interpretation of findings. A scoping review will be used to map existing evidence and describe how postpartum nerve injury following epidural analgesia has been investigated.

The objective of this scoping review is to map the methodological approaches used in studies of postpartum nerve injury, including case identification, diagnostic assessment, follow‐up procedures, and attribution of obstetric and anesthetic factors. The findings aim to inform the development of a methodological template for future trials.

## 2 Methods

### 2.1 Review Questions

This scoping review will aim to address the following review questions:

1. How are women with postpartum nerve injury identified and recruited in these studies, including inclusion pathways and reporting sources?
2. What definitions and algorithms are used to define and classify postpartum nerve injuries across studies?
3. Which diagnostic methods are applied to assess and confirm postpartum nerve injury?
4. How are recovery and follow‐up processes described and monitored?
5. How are obstetric and anesthetic risk factors reported in studies of epidural analgesia during vaginal birth, and what approaches are used to differentiate obstetric neuropathy and neuraxial‐related injury?

The questions are intended to guide study selection, data charting, and synthesis of methodological characteristics.

### 2.2 Eligibility Criteria

Eligibility criteria will be defined based on the review questions and structured according to the Population‐Concept‐Context (PCC) framework [10].

The population will be defined as women aged 18 years and older who have undergone vaginal birth with epidural analgesia. The concept of interest will include postpartum neurological symptoms or deficits suggestive of peripheral nerve injury identified after delivery. The context will be limited to hospital‐based settings where epidural analgesia was used. Studies including mixed modes of delivery will be excluded unless results are reported separately for vaginal births with epidural analgesia. Studies including mixed anesthesia will be excluded if epidural cases cannot be extracted.

Eligible studies will include prospective and retrospective study designs. Studies must report at least one of the following: the occurrence or type of postpartum nerve injury, diagnostic or assessment methods, timing of symptom onset, or recovery and follow‐up patterns. Studies must include more than 50 patients.

Case reports, case series, reviews, expert opinions, and studies without data on postpartum neurological symptoms will be excluded.

Manuscripts published in all languages will be eligible for inclusion. Articles published in languages other than Danish, English, Norwegian, or Swedish will be translated using AI‐assisted translation tools. Publications from 1990 onwards will be eligible to reflect modern obstetric anesthesia practices, equipment, and safety standards [2].

The eligibility criteria that will be used during the screening process are provided in Table A1.

### 2.3 Search Strategy and Information Sources

Two articles on obstetric neuraxial complications [7, 11] will support development of the search framework and selection of search terms. The articles will guide the identification of relevant complications, study designs, and outcome domains used in the search strategy.

A systematic search will be conducted in MEDLINE, EMBASE, PubMed, and the Cochrane Library in collaboration with an information specialist. An initial pilot screening of the first 200 records retrieved will be performed independently by two reviewers to assess whether the eligibility criteria identify relevant studies. If needed, the search strategy or eligibility criteria will be revised and pilot screening repeated until the final search strategy is established.

Reference lists of included studies will be screened to identify additional relevant publications. The PubMed search strategy is provided in Table A2. Searches in EMBASE, MEDLINE, and Cochrane Library will follow an equivalent structure adapted to each database.

### 2.4 Selection of Studies

All records retrieved through the database searches will be imported into Covidence [12] for duplicate removal and systematic screening. Study selection will be conducted in two stages: title and abstract screening followed by full‐text screening.

Two reviewers will independently screen titles and abstracts using the predefined eligibility criteria. Potential eligible full‐text articles will be assessed independently by the same two reviewers. Any disagreements concerning study eligibility will be resolved through discussion, and if consensus cannot be reached, an arbitrator will be consulted.

The study selection process will be documented using a PRISMA flow diagram, visualizing the number of articles identified, screened, excluded, and included at each stage.

### 2.5 Data Charting Process

Data charting will use predefined templates based on the review questions. The templates will be reviewed and refined by two reviewers as data charting progresses. Data will be extracted as reported in the original studies. Any disagreements will be resolved through discussion or, if needed, consultation with an arbitrator. Extracted data will include basic study characteristics (author, year, country, design, setting, and sample size); methodological information will be organized according to the review questions.

Baseline characteristics and pre‐pregnancy neurological status will be charted. Data on case identification and recruitment will include inclusion pathways, timing of recruitment, and reporting sources. Case identification method will be recorded and grouped into clinician‐initiated, patient‐initiated, or registry‐based.

Data charting on the definition and classification of postpartum nerve injury will include the terminology used in individual studies, diagnostic definitions, and any classification systems applied. Information on anatomical localization and associated functional symptoms will also be recorded (e.g., mononeuropathies, plexopathies, radiculopathies, or central neuraxial involvement).

Data on diagnostic assessment will be extracted on neurological examination, including how examinations are described and performed. Information on use of additional diagnostic modalities will be extracted, including imaging and electrophysiological testing. Timing of electromyography or nerve conduction studies will be charted.

Recovery and follow‐up data will be charted, including follow‐up structure, duration, responsible personnel, and outcome measures. Questionnaires, interview guides, or algorithms used in the included studies to assess symptom resolution or persistence will also be charted.

Data will be extracted on obstetric and anesthetic risk factors to address the fifth research question on exposure reporting and attribution of causation.

Obstetric variables will include: mode of vaginal delivery, type of instrumental delivery, duration of the second stage of labor, maternal positioning, fetal weight or macrosomia, fetal malposition, parity, and other factors described in relevant literature [5].

Anesthetic variables will include: epidural technique, placement details, initial effect and other relevant factors retrieved from literature found through the search [5]. Information on attribution of nerve injury to obstetric or neuraxial causes will be charted.

Missing or unclear information will be documented as reported in the original studies.

### 2.6 Synthesis of Result

Extracted data will be synthesized descriptively to address the predefined review questions. Consistent with PRISMA‐ScR guidance for scoping reviews, the synthesis will take a broad approach to map the variability of methodology in the literature.

The synthesis will follow the same methodological domains used in the data charting process. The domains will include case identification and inclusion pathways, definitions and classification of postpartum nerve injury, diagnostic assessment methods, recovery and follow‐up processes, and reporting of obstetric and anesthetic exposures and attribution of causation.

Findings will be presented in structured tables and narrative summaries to illustrate similarities, differences, and gaps in methodological approaches across studies.

## 3 Discussion

This scoping review will systematically map how postpartum nerve injury following labor epidurals has been defined, identified, assessed, and followed in the literature, with the aim of clarifying methodological patterns and inconsistencies across studies.

The findings may inform future research by highlighting variation in methodology, reporting practices, and terminology. In particular, the results may support the development of more consistent approaches to neurological assessment, exposure reporting, and attribution of obstetric and anesthetic factors. Based on the findings of this review, a methodological template for future trials will be developed and presented.

## Author Contributions

Authorship follows the criteria of the International Committee of Medical Journal Editors [13]. Louise Mathilde Loft Sakhat developed the protocol and wrote the initial draft. Kim Wildgaard contributed to the conception and design of the protocol and critically reviewed the manuscript as primary advisor. Amanda Prip Dohn, Bo Biering‐Sørensen, Kenneth Geving Andersen, and Line Thellesen contributed to the critical revision of the manuscript and approved the final version.

## Funding

No financial funding has been granted for this manuscript.

## Conflicts of Interest

The authors declare no conflicts of interest.

## Data Availability

No new data was generated in this review, and therefore data sharing is not applicable.

## Appendix A

**TABLE A1:** Eligibility Criteria.

Criterion	Inclusion	Exclusion
Population	Women ≥ 18 years, vaginal birth	Non‐obstetric populations Cesarean section only
Exposure	Epidural analgesia used during labor	Spinal anesthesia only General anesthesia only Epidural exposure not reported or cannot be separated
Outcome	Postpartum neurological evaluation, symptoms, neurological deficits, or peripheral nerve injury after delivery	Studies reporting no neurological outcomes
Concept	Neurological outcomes/peripheral nerve injury (e.g., neuropathy, neurological deficit, sensory or motor disturbance)	Outcomes unrelated to neurological complications
Context	Hospital‐based obstetric settings where epidural analgesia is administered	Non‐clinical settings or studies not related to obstetric care
Study design	Observational studies (prospective or retrospective), cohort studies, case–control studies, cross‐sectional studies, registry studies, randomized controlled trials	Case reports, case series, narrative reviews, systematic reviews, editorials, expert opinions, guidelines
Sample size	Studies including more than 50 participants	Studies with less than 50 participants
Publication year	Published 1990 onwards	Published before 1990
Language	All languages eligible	—
Mixed delivery modes	Studies including multiple delivery modes if results for vaginal birth with epidural can be extracted separately	Studies with mixed delivery modes if vaginal epidural cases cannot be separated
Mixed anesthesia	Studies including multiple anesthesia techniques if epidural data can be extracted separately	Studies with mixed anesthesia if epidural cases cannot be separated

*Note:* Eligibility criteria used during the screening process for the scoping review: Nerve injury following birth, the role of epidural analgesia.

Abbreviation: PCC, population, concept, context framework (Pollock et al. [10]).

**TABLE A2:** PubMed search strategy.

Search	Concept	Search string	Results
#1	Population—free‐text terms	(pregnant[tiab] AND (woman[tiab] OR women[tiab])) OR pregnan*[tiab] OR obstetric*[tiab] OR parturition[tiab] OR labor[tiab] OR labour[tiab] OR childbirth*[tiab] OR “vaginal birth*”[tiab] OR “vaginal deliver*”[tiab] OR (deliver*[tiab] AND obstetric*[tiab]) OR postpartum[tiab]	935,066
#2	Population—MeSH terms	“Labor, Obstetric”[Mesh] OR “Parturition”[Mesh]	71,772
#3	Population—additional free‐text	parturition[tiab] OR ((woman[tiab] OR women[tiab]) AND (labor[tiab] OR labour[tiab])) OR childbirth*[tiab]	84,802
#4	Population—combined	#1 OR #2 OR #3	954,845
#5	Outcome—free‐text terms	“nerve injur*”[tiab] OR “Peripheral Nervous System Disease*”[tiab] OR neuropath*[tiab] OR (nerve[tiab] AND damage*[tiab]) OR (paresthesia[tiab] OR paraesthesia[tiab]) OR “sensory loss*”[tiab] OR (hypoesthesia[tiab] OR hypoesthaesia[tiab]) OR “nerve lesion*”[tiab] OR “sensorimotor* deficit*”[tiab] OR “High neuraxial block”[tiab] OR (neurolog*[tiab] AND deficit*[tiab])	317,960
#6	Outcome—transient/mild qualifiers	(transient[tiab] OR temporary[tiab] OR mild[tiab] OR reversible[tiab]) AND (“nerve injur*”[tiab] OR neuropath*[tiab] OR “neuro‐path*”[tiab] OR “neurologic* deficit*”[tiab] OR “nerve damage*”[tiab] OR plexopathy[tiab])	24,465
#7	Outcome—neurological assessment	“neurologic* exam*” OR “neurologic* eval*”	48,633
#8	Outcome—combined	#5 OR #6 OR #7	357,636
#9	Exposure—free‐text terms	((anesth*[tiab] OR anaesth*[tiab]) AND epidural*[tiab]) OR ((anesth*[tiab] OR anaesth*[tiab]) AND obstetric*[tiab]) OR ((epidural[tiab] OR regional[tiab]) AND (anesth*[tiab] OR anaesth*[tiab])) OR ((spinal[tiab] OR neuraxial[tiab]) AND (anesth*[tiab] OR anaesth*[tiab])) OR ((spinal[tiab] OR neuraxial[tiab]) AND procedure*[tiab]) OR (epidural[tiab] AND analgesia[tiab]) OR (obstetric*[tiab] AND analgesia[tiab]) OR (“labor epidural*”[tiab] OR “labour epidural*”[tiab]) OR (obstetric*[tiab] AND epidural*[tiab]) OR epidural*[tiab] OR (neuraxial[tiab] AND intervention*[tiab]) OR (regional[tiab] AND block*[tiab])	140,635
#10	Exposure ‐ MeSH terms	“Analgesia, Epidural”[Mesh] OR “Analgesia, Obstetrical”[Mesh] OR “Anesthesia, Obstetrical”[Mesh]	24,519
#11	Exposure—conduction anesthesia	“Anesthesia, Conduction”[Mesh] OR conduction anesthesia[tiab]	76,466
#12	Exposure—combined	#9 OR #10 OR #11	190,966
#13 (Final)	Final search: Population AND Outcome AND Exposure	#4 AND #8 AND #12	520

*Note:* Search conducted: 24 March 2026.

Abbreviations: Mesh, medical subject headings; tiab, title/abstract field.
